# Chromatin Methylation Abnormalities in Autosomal Dominant Polycystic Kidney Disease

**DOI:** 10.3389/fmed.2022.921631

**Published:** 2022-07-05

**Authors:** Jing Xu, Cheng Xue, Xiaodong Wang, Lei Zhang, Changlin Mei, Zhiguo Mao

**Affiliations:** ^1^Kidney Institute, Department of Nephrology, Shanghai Changzheng Hospital, Second Military Medical University, Shanghai, China; ^2^State Key Laboratory of Cell Biology, Center for Excellence in Molecular Cell Science, Shanghai Institute of Biochemistry and Cell Biology, Chinese Academy of Sciences, University of Chinese Academy of Sciences, Shanghai, China; ^3^School of Life Science and Technology, Shanghai Tech University, Shanghai, China; ^4^School of Life Science, Hangzhou Institute for Advanced Study, University of Chinese Academy of Sciences, Hangzhou, China

**Keywords:** DNA methylation, histone methylation, RNA methylation, autosomal dominant polycystic kidney disease, epigenetics

## Abstract

Autosomal dominant polycystic kidney disease (ADPKD) is the most common inherited kidney disease worldwide and is one of the major causes of end-stage renal disease. *PKD1* and *PKD2* are two genes that mainly contribute to the development and progression of ADPKD. The precise mechanism is not fully understood. In recent years, epigenetic modification has drawn increasing attention. Chromatin methylation is a very important category of PKD epigenetic changes and mostly involves DNA, histone, and RNA methylation. Genome hypomethylation and regional gene hypermethylation coexist in ADPKD. We found that the genomic DNA of ADPKD kidney tissues showed extensive demethylation by whole-genome bisulphite sequencing, while some regional DNA methylation from body fluids, such as blood and urine, can be used as diagnostic or prognostic biomarkers to predict PKD progression. Histone modifications construct the histone code mediated by histone methyltransferases and contribute to aberrant methylation changes in PKD. Considering the complexity of methylation abnormalities occurring in different regions and genes on the PKD epigenome, more specific therapy aiming to restore to the normal genome should lead to the development of epigenetic treatment.

## Introduction

Autosomal dominant polycystic kidney disease (ADPKD) is the most common form of inherited kidney disease and is one of the major causes of end-stage kidney disease (ESKD), affecting 1 in 2,500 to 1,000 individuals worldwide (1, 2). The mutation in either of two genes mainly contributed to the development and progression of ADPKD (3): *PKD1* and *PKD2. PKD1* encodes a large, multidomain integral membrane protein, polycystin-1 (PC1) (4), and *PKD2* encodes a calcium ion channel of the transient receptor potential family, polycystin-2 (PC2) (5, 6). The *PKD1* gene mutation accounts for approximately 80% of ADPKD cases in clinically identified populations, while *PKD2* gene mutation is responsible for approximately 10% of cases, and GANAB, DNAJB11, ALG9, IFT140, etc. account for the rest (7). Defects in the expression or function of PC1 or PC2 result in cystogenesis; however, the precise mechanism has not been fully understood, although numerous studies have been carried out on the functional roles of PC1 and PC2 and their downstream effector pathways.

The phenotype and progression of ADPKD are highly variable, irrespective of mutations in the same *PKD* genes (8). Even in the same family or with an identical gene mutation background, the disease course and rate of progression to renal failure are diverse, suggesting that there are factors in addition to gene mutations that influence patient prognosis (9). The molecular mechanism for the variability still remains unsolved, but epigenetic modification might be responsible and has drawn increasing attention in recent years (10).

Epigenetics focuses on genome-wide changes in gene expression or phenotype caused by DNA methylation, non-coding RNA modifications, histone modifications, including acetylation, methylation, and ubiquitination (10–12). Similar to the arrangement of DNA sequences, epigenetic information can also be inherited, and its activation and silencing are the switches that turn genes on and off (13, 14). It also provides clues to the interpatient variabilities of disease progression and response to treatment (12). Chromatin methylation is a very important category of PKD epigenetic changes and mostly involves DNA methylation and histone methylation; moreover, RNA methylation has aroused great interest recently (15). Recent evidence suggests that alterations of DNA and histone methylation, as well as RNA methylation on specific genes and the whole genome, contribute to the pathogenesis of PKD.

## DNA Methylation and ADPKD

DNA methylation is a key epigenomic feature that controls the suppression and expression of genes by the addition of a chemical methyl group mediated by DNA methyltransferases (DNMTs) (16). DNMT1, as the maintenance methyltransferase, binds mainly to hemimethylated DNA during DNA replication and is responsible for accurately replicating DNA methylation patterns during the S phase of the cell cycle, while *de novo* methylation is preferentially mediated by DNMT3a and DNMT3b (16). DNA methylation in the whole genome and at specific loci can be quantified across the entire genome in a sequence-specific manner to generate a methylome map and can be quantified in either circulating free DNA or single cells.

DNA methylation commonly emerges at cytosine-guanosine dinucleotides (CpGs), where the methyl group is added to the fifth carbon of the cytosine, forming 5-methylcytosine (17). Approximately 10% of human genomes contain CpG sites. DNA is methylated at approximately 75% of all CpG sites in mammalian genomes, primarily in heterochromatic regions (18). Clusters of grouped CpGs called CpG islands are often located near the promoter or enhancer regions of human genes. Methylation within a gene promoter has usually been considered a repressor of the gene by reducing the binding ability of transcription factors. One mechanism for transcription factor inhibition by methylation is chromatin remodeling, as there is epigenetic crosstalk between methylation and histone modification (18). However, methylation of CpGs within the gene body is sometimes controversial compared with promoter methylation, which typically results in increased or sustained gene expression. Only ~2% of regions of DNA are rigorously protected from methylation and are associated with transcription start sites in almost half of human genes. Possible mechanisms have been proposed: methylated regions contain genomic elements responsible for alternative splicing, containing transcription factors interfering with host gene expression when hypomethylated, or residual genomic imprinting developed from embryonic stages (18). Moreover, several studies have shown that the hypermethylation of gene bodies can silence gene expression, especially in highly expressed genes (18).

### Genome-Wide Platforms to Detect DNA Methylation in ADPKD

**Figure 1:**
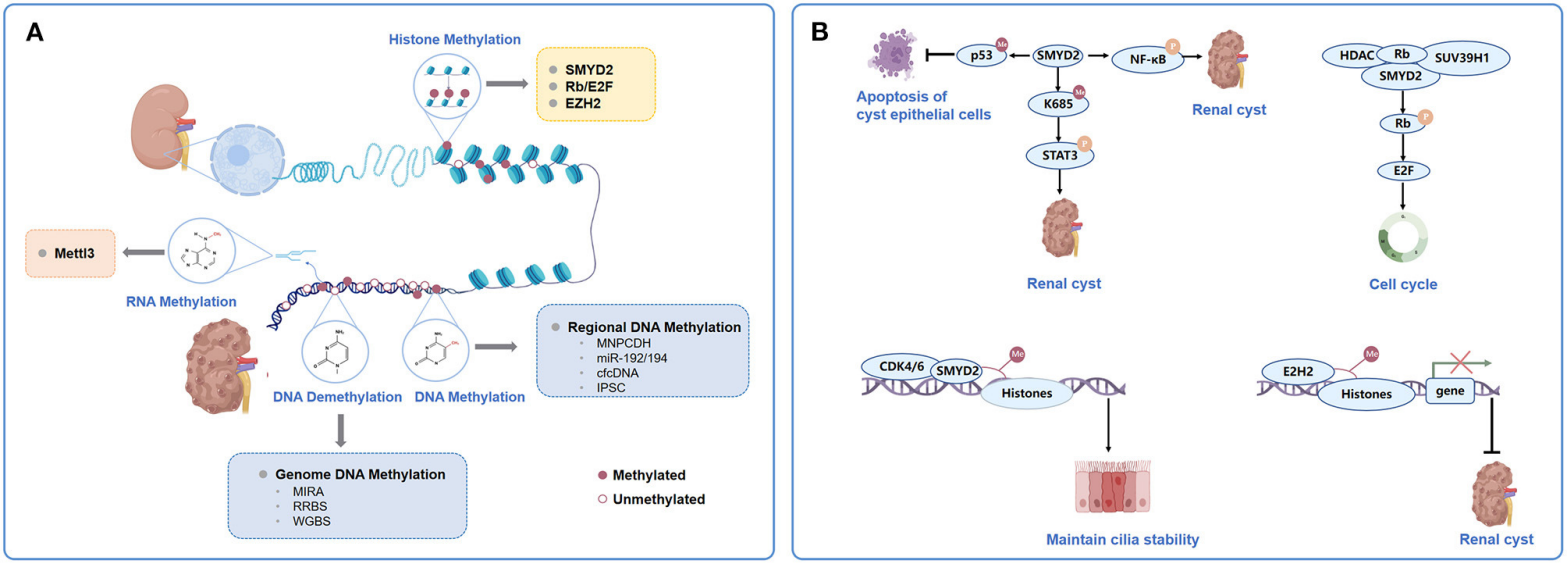
Major types and mechanism of chromatin methylation in ADPKD. **(A)**. Categorization of chromatin methylation in ADPKD. The chromatin methylation in ADPKD mostly involves DNA methylation, histone methylation and RNA methylation. Genome-wide platforms to detect DNA methylation in ADPKD including MIRA, RRBS and WGBS. Genome hypomethylation and gene hypermethylation coexist in ADPKD. Regional DNA methylations could predict application as biomarkers in ADPKD, such as methylation of MUPCDH, miR-192/194, cfcDNA and iPSC. Histone methylations were mediated by HMTs. Some of the mediators and modulators to be closely related to the pathogenesis and acceleration of cystic phenotypes together with or acting as the HMTs, mainly represented by SMYD2, Rb/E2F and EZH2. The adenosine methylation at the 6th nitrogen position known as m6A, mediated by the multi-protein m6A writer complex composed of Mettl3, was involved in pathogenesis of mouse and human ADPKD. **(B)** Participation of SMYD2, Rb/E2F and EZH2 in histone methylation in ADPKD. ADPKD, autosomal dominant polycystic kidney disease; MIRA, methylated- CpG Islandisland recovery assay; RRBS, reduced representation bisulfitebisulphite sequencing; WGBS, whole-genome bisulfitebisulphite sequencing; MUPCDH, mucin-like protocadherin; cfcDNA, cell-free circulating DNA; iPSC, induced pluripotent stem cells; HMT, histone methyltransferases; Rb,Retinoblastoma tumourtumor suppressor protein.

The first technology used in ADPKD research was the methylated CpG island recovery assay (MIRA) (Figure 1A) (19). The principle of this method is that the methylation-CpG-binding protein MBD2 specifically recognizes and binds to the methylation sequence (20). The MBD3L1 protein can enhance the binding ability of MBD2 to methylated CpG. Based on this principle, different DNA fragments with different methylation levels are separated by the chromatographic column method according to the different retention times of DNA bound to MBD2 in the chromatographic column (21). After connecting these DNA fragments to oligonucleotide junctions, the whole genome was amplified by PCR, purified, hybridized with an Agilent chip, and sequenced by the Agilent platform. This technique can find highly methylated regions in the genome, but it cannot analyse methylation at a single base level (resolution is approximately 150 bp). It can only judge whether methylation exists in a certain region by the enrichment peak. It also cannot quantify the level of methylation. Moreover, the detection of this technique has an obvious tendency. It can only detect areas with high CpG density and methylation (22).

Four years later, Bowden et al. (23) used reduced representation bisulphite sequencing (RRBS) to detect the genomic DNA methylation of ADPKD. However, RRBS only sequenced a reduced, representative sample of the whole genome (approximately 1% of the genome region) (24). For epigenetic therapy to be applied to ADPKD, we need to obtain the specific distribution of methylated CpG across the genome, not just globally or selectively (25).

Whole-genome bisulphite sequencing (WGBS) is currently considered the gold standard in DNA methylation profiling (24), potentially allowing the investigation of every CpG site in the genome. WGBS can provide single-base resolution with full genome coverage without the biases associated with selecting agents (26). As next-generation sequencing costs decrease, WGBS has become increasingly accessible for clinical research. These methods could profile different areas of the genome based on a design to identify important regions and reveal DNA methylation changes in *PKD1*.

### Genome DNA Methylation in Human ADPKD

DNA methylation is a key epigenetic modification that plays a critical role in the modulation of gene expression in diseases, especially cancers (27, 28). Global hypomethylation is one of the major characteristics of cancer; however, focally hypermethylated DNA methylomes also coexist in cancers, which are usually located at CpG islands and are closely associated with promoter activity and gene expression (29). This also explains the theory of using methyltransferase inhibitors to treat cancers (13).

As a tumor-like disease, ADPKD might theoretically share similar DNA methylation properties with cancers (30). To date, the characteristics of DNA methylation in ADPKD remain inconclusive (Table 1). In 2014, Woo et al. (31) reported global DNA methylation levels in human ADPKD for the first time. This study was conducted on kidneys from 3 ADPKD patients compared with 3 non-ADPKD samples by pyrosequencing. This study found that 91% of over 13,000 unique fragments of the genome in ADPKD exhibited hypermethylation, mainly at exonic regions. Woo et al. also found hypermethylation of exon 43 in *PKD1* gene-body regions, along with silencing of *PKD1* expression, which is involved in cystogenesis (31). Moreover, demethylation of an ADPKD cell line (WT 9–12) resulted in increased *PKD1* expression, and treatment with a DNMT inhibitor repressed the cyst growth of the MDCK cyst-forming cell line. In 2018, Bowden et al. (23) observed approximately 2% global hypomethylation of the genome in ADPKD compared with non-ADPKD kidneys using RRBS for genome-wide methylation analysis. The *PKD1* gene body was hypermethylated in ADPKD by the RRBS method, but Bowden et al. (23) found that hypermethylation was associated with an increase in *PKD1* expression rather than a decrease. However, Hajirezaei et al. (32) found that the *PKD1* promoter was hypomethylated and was inversely correlated with *PKD1* expression in patient blood, further indicating the controversial role of DNA methylation in *PKD1* expression. To further investigate the epigenetic mechanism of discrepant developments of renal cysts in the same ADPKD context, a genome-wide DNA methylation analysis by Bowden et al. (33) included eight renal cysts from one ADPKD patient. The results showed that 14.6% of the analyzed fragments exhibited a large amount of intercyst DNA methylation variants. Fragments in CpG islands and gene bodies harbored most of the methylation variations across each cyst, while intergenic fragments were comparatively stable. The epigenetic variation overlapped with the transcriptional activity in ADPKD. This study showed the global methylation patterns of individual cysts.

**Table 1:** Summary of DNA methylation analysis in ADPKD.

**Study**	**Year**	**Platform**	**Number**	**Sample Type**	**ADPKD overall**	***PKD1* gene**	**PKD1 expression**
Woo	2014	MIRA-Seq	6	Kidney	Hypermethylation	Hypermethylation in gene-body	Decreased
Bowden	2018	RRBS	3& non-ADPKD	Kidney	Hypomethylation	Hypermethylation in gene-body	Increased
Bowden	2020	RRBS	8 cysts from 1 ADPKD	Kidney	Hypomethylation in cysts	NA	NA
Hajirezaei	2021	MS-HRM	80	Kidney	NA	PKD1 promoter hypomethylation	Increased
Our study	2022	WGBS	10	Kidney	Hypomethylation	Hypomethylation in gene body and promoter	NA

*NA, not available*.

**Figure 2:**
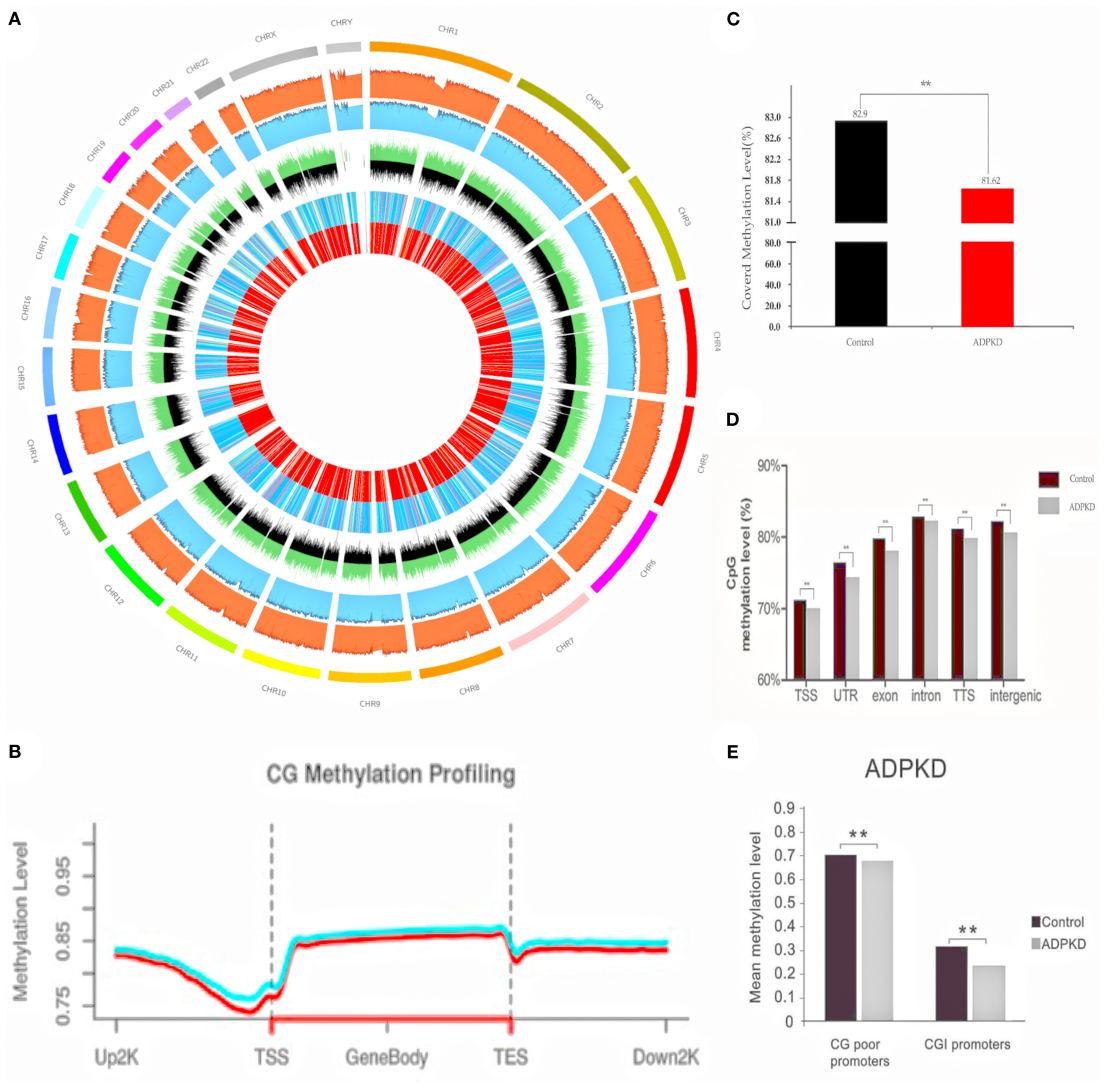
WGBS of five non-ADPKD and five ADPKD kidneys. **(A)** Circos representation of genome-wide DNA methylation levels in the non-ADPKD and ADPKD groups. Average levels for all of the CGs in 29710-Mbp windows. The inner track indicates the magnitude of the difference between the non-ADPKD and ADPKD groups for each window (color scale and red line). Average methylation levels in all of the regions are expressed as β-values (0–1) and are colored blue. **(B)** CG methylation profiling graph for the non-ADPKD group (green line) and the ADPKD group (red line). **(C)** Methylation level of covered CpG in the non-ADPKD group and the ADPKD group (rank-sum test, ** *p* < 0.01). **(D)** Mean CpG methylation levels among different genomic sequences in the non-ADPKD group and the ADPKD group (rank-sum test, ** *p* < 0.01). **(E)** Mean CpG methylation levels among promoters in the non-ADPKD and ADPKD groups in the presence or absence of a CpG island (rank-sum test, ** *p* < 0.01). The mean methylation level was calculated by the number of total methylated reads divided by the number of total reads covering CpG sites located in the sum of the features analyzed.

We used WGBS to examine the ADPKD DNA methylome in humans (34). DNA was extracted from the renal cortex tissues of five ADPKD patients and five non-ADPKD patients with renal cell carcinoma (cortex tissue far away carcinoma). We generated ~33 × 10^7^ 150 bp paired-end reads corresponding to the global coverage of ~30 × sequencing depth in WGBS and successfully mapped ~80% of the genome for each sample. The full set of WGBS data from ADPKD and non-ADPKD samples is illustrated in Figure 2A using Circos. CpG methylation profiling is shown in Figure 2B. The mean methyl CpG levels of the two groups are shown in Figure 2C (rank-sum *test, p* < 0.0001). ADPKD showed global hypomethylation compared with non-ADPKD. For functional genomics, we observed methylated CpGs on various genomic compartments, such as the transcriptional start site (TSS, ± 2 kb around the transcription start site) and intronic, exonic, and intergenic regions (for each region: rank-sum *test, p* < 0.0001, Figure 2D). The short (approximately 1 kb) CpG-rich regions, known as CpG islands, are often located within and close to sites of approximately 40% of promoters. After subclassifying the promoters according to their CpG density, we found that ADPKD DNA exhibited more unmethylated CpGs, especially at CpG-poor promoters, than non-ADPKD samples (Figure 2E). We confirmed that hypermethylation was not enriched in CpG islands in ADPKD, which was different from cancer (35). Overall, we found that the genomic DNA of human ADPKD kidney tissues showed extensive demethylation; however, the overall hypomethylation of the ADPKD genome could not exclude the possibility that a few areas may be hypermethylated, some of which could even predict disease progression and prognosis (36). More studies are needed to investigate the influence and functional changes of methylation diversity, mainly focusing on genome hypomethylation, in ADPKD pathogenesis.

### Regional DNA Methylations as Biomarkers in ADPKD

Genome hypomethylation and gene hypermethylation coexist in ADPKD. DNA methylation from body fluids, such as blood and urine, can be detected and quantified as diagnostic or prognostic biomarkers in ADPKD (Figure 1A) (12, 37). We could predict their application as potential markers in ADPKD for renal cyst development, altered renal function, and ultimately disease progression. Mucin-like protocadherin (MUPCDH) is a novel member of the cadherin superfamily, which is especially expressed at the apical surface of differentiated proximal tubule epithelial cells of the kidney (36, 38). Methylated CpG island recovery assay-DNA sequencing (MIRA-seq) analysis detected the DNA methylation pattern of the MUPCDH gene promoter region (36). Further study showed that in random urine samples from ADPKD, patients with a fully methylated MUPCDH promoter had a higher percent annual change in total kidney volume (TKV), reflecting the faster progression of cyst growth. Urine methylated-modified MUPCDH might be a prognostic biomarker for ADPKD. Whether the methylation status of urinary genomic DNA can predict cyst development and loss of renal function in ADPKD requires further investigation.

As epigenetic regulators, microRNAs (miRs) are endogenous, small, non-coding RNAs that are ~22 nt in length and could be used to predict disease progression and patient prognosis (39). Genome-wide analyses of miR expression and DNA methylation status in ADPKD showed that two members of the miR-192 family, miR-192 and miR-194, displayed greater enrichment of the methylated DNA fraction and diminished expression in ADPKD (38). They can affect cyst enlargement by targeting endothelial-mesenchymal transition (EMT)-related genes, such as zinc finger E-box-binding homeobox-2 (ZEB2) and cadherin-2 (CDH2).

Cell-free circulating DNA (cfcDNA) in blood has been reported to carry distinctive DNA methylation markers in certain GC-rich fragments, which comprise CpG islands (40). Differential DNA methylation analysis of cfcDNA has been used successfully for prenatal diagnosis with applications for cancer diagnosis and monitoring of treatment efficacy (41), which might provide specific and sensitive potential epigenetic signatures for diagnosis, prognosis and prediction of response to therapy in ADPKD.

Finally, Gribnau et al. (14) generated induced pluripotent stem cells (iPSCs) from ADPKD patients. The cell whole-genome DNA methylation analysis (MeD-seq) showed that cystic epithelium-derived iPSCs maintained kidney-specific DNA methylation memory. Gene ontology (GO) analysis with PKD-specific hypermethylated gene body differentially methylated regions (DMRs) retrieved gene ontology (GO) terms, such as cell–cell adhesion and cell–cell signaling, which were retained as epigenetic signatures.

## Histone Methylations in ADPKD

Aberrant methylation changes in PKD also correlate with histone modifications, which are the second epigenetic mechanism that regulates gene expression and protein function (Figure 1A) (12). They can lead to either gene activation or repression depending on which histone residue is modified (11). Park et al. (31) performed histone ChIP–qPCR in ADPKD patients and found that active histone methylation marks, viz. H3K36me3 and H3KAc increased significantly in the *Pkd1* gene-body region, whereas a repressive histone modification mark (H3K27me3) decreased. Modifications of these residues were associated with the elimination of DNA methylation in *Pkd1*.

Histone methylation is mediated by histone methyltransferases (HMTs), which are epigenetic modifiers that directly modify the epigenome (12). There are two families of HMTs, which have specificities for arginine and lysine residues (42). In PKD, they are located upstream of epigenetic mediators, which are the direct targets of them or their downstream targets of epigenetic modification; meanwhile, they are downstream of epigenetic modulators, which influence the activity or localization of the epigenetic modifiers and the epigenetic states mediated by them (43). Some of the mediators and modulators have been reported to be closely related to the pathogenesis and acceleration of cystic phenotypes together with or acting as the HMTs (44). They form covalent modifications on the N-terminal tails of PKD genes, which are rich in histone residues, and construct the histone code together through their interacting arrangements.

### SMYD2

SMYD2 is a SET and MYND (myeloid-Nervy-DEAF1) domain protein (45). It acts as a lysine methyltransferase to regulate cyst growth in ADPKD through multiple signaling pathways (Figure 1B) (46–48). First, SMYD2 acts as a downstream mediator of Pkd1 mutation and activates phosphorylated STAT3 in JAK/STAT signaling, which serves as a positive regulator of cyst growth, via lysine methylation at K685 (48). It can also promote cystic renal epithelial cell proliferation and survival by methylating the subunit of NF-κB, p65 at lysine 310 and partially at lysine 221, leading to its phosphorylation and activation (48). Moreover, SMYD2 could integrate epigenetic regulation and renal inflammation in cyst development through the formation of two positive feedback loops: SMYD2/IL-6/STAT3/SMYD2 and SMYD2/TNF-α/NF-κB/SMYD2 (48). Furthermore, it could increase the methylation of p53 and prevent p53-dependent cystic renal epithelial cell apoptosis (45, 48). Double conditional knockout of the Pkd1 and Smyd2 genes in a PKD mouse model delayed renal cyst growth and preserved renal function (48). Specifically, inhibiting Smyd2 could slow disease progression, indicating that it is a novel therapeutic target for ADPKD treatment.

Further study showed that epigenetic histone modifications by SMYD2 were also involved in the regulation of the cell cycle and ciliagenesis in ADPKD (47). SMYD2 was found to be colocalized at the basal body of the primary cilia together with cyclin-dependent kinase 4 (CDK4) and its closely related CDK6 (49). CDK4/6 can form complexes with D-type cyclins and drive G1 phase quiescent cells into DNA synthesis S phase (50). CDK4/6 interacts with SMYD2 and regulates the methylation of histone H3 lysine 4 (H3K4) and lysine 36 (H3K36), which promote the phosphorylation and enzymatic activity of SMYD2; on the other hand, methylation of histone H3K4 at the promoters of CDK4 and CDK6 by SMYD2 positively regulates their transcription (47). CDK4/6-SMYD2 signaling maintained the balance of microtubule dynamics through methylation of the key components affecting cilia assembly: (i) it methylated α-tubulin at lysine-394 (TubK394me) and retarded the stability of microtubules, which facilitated the trafficking of cilia proteins from the Golgi to the ciliary base; (ii) it also methylated histone H3K36 at the promoter of intraflagellar transport protein IFT20, which encodes a key IFT protein that regulates the trafficking of ciliary proteins from the Golgi to cilia, to repress its transcription. Depletion or inhibition of CDK4/6-SMYD2 signaling selectively decreased the methylation of α-tubulin and increased the expression of IFT20, resulting in an improved fidelity in the number of ciliated cells and cilia length, which might contribute to the slowing down of cystic renal epithelial cell proliferation and cyst growth (47).

### Rb/E2F

Retinoblastoma tumor suppressor protein (Rb)/E2F could be both epigenetic mediators and modulators in cyst development in ADPKD (Figure 1B) (10). The hypophosphorylated state Rb acted as a repressor of E2F-mediated transcriptional activity, which could form a complex with histone deacetylases (HDACs), DNMT1 and SUV39H1, an HMT that recruits them to E2F-site-containing promoters, including cyclin A/E and Cdk2 (51). SMYD2 has been reported to methylate Rb at lysine 810, which enhances Ser807/811 phosphorylation of the Rb protein. This accelerates E2F transcriptional activity and promotes cell cycle progression (46). HDAC inhibition targeting Rb/E2F has been reported to decrease cystic epithelial cell proliferation and reduce cyst development by acting as an inhibitor of differentiation 2 (Id2) (52) and sirtuin 1 (53), respectively; however, its influence on histone methylation in ADPKD cystogenesis requires further investigation.

### EZH2

The polycomb group (PcG) gene EZH2, also known as the homolog of Enhancer of zeste in mammals, is a histone modifier that plays a crucial role in differentiation gene silencing in a cell cycle-dependent manner (54). It is the major methyltransferase for H3 lysine 27 and plays a crucial role in differential gene silencing, such as for HOX genes and SOX family members (55). It is also the direct target of the core cell cycle transcriptional regulator E2F (56). EZH2 is upregulated in cystic renal epithelial cells, the targeting of which delayed cyst growth in Pkd1 knockout mouse models (Figure 1B) (12). Further investigations will be required to fully understand how elevated EZH2 coordinates with the cell cycle machinery, such as Rb/E2F, to promote cystic cell proliferation.

## RNA Methylation

Modifications of RNA can change its processing, stability, or translational efficiency, similar to DNA (57). The methylation of RNA has been a recently discovered aspect of epigenetic modification in ADPKD (15). Adenosine methylation at the 6th nitrogen position known as N^6^-methyladenosine (m^6^A), recognized as the most common eukaryotic RNA modification and mediated by the multiprotein m^6^A writer complex composed of Mettl3, etc., was increased in mouse and human ADPKD samples (Figure 1A) (15, 58). Kidney-tubule-specific Mettl3 overexpression induced the occurrence and enlargement of tubular cysts, and conversely, Mettl3 deletion attenuated cyst growth in three different orthologous ADPKD transgenic mouse models, irrespective of the type of *PKD1* mutation or dynamics of cyst growth (15). Further immunoprecipitation of m^6^A-modified mRNAs and high-throughput sequencing (MeRIP-seq) showed that c-Myc and Avpr2 mRNA had significantly higher m^6^A modification and translation, which enhanced c-Myc and Avpr2 protein expression and resulted in cyst proliferation and fluid protein synthesis through the c-Myc and cAMP signaling pathways. Moreover, dietary methionine and S-adenosylme thionine, which could induce Mettl3 expression, aggravated cyst growth *ex vivo* (59); in contrast, dietary methionine restriction attenuated mouse ADPKD, indicating a potential dietary therapy to slow down disease progression (15, 60).

## Epigenetic Therapy in ADPKD and Future Perspectives

Unlike genetic mutation, epigenetic silencing is a potentially reversible alteration, which means that through optimal epigenetic therapy, it can be restored to the normal status and used as a treatment for ADPKD (61). For example, restoring the diminished expression of miR-192 and miR-194 by injection of their precursors to replace the hypermethylated non-functional ones could reduce the size of cysts in a Pkd1 knockout mouse model (38). Restoration of MUPCDH expression by using 5-azacytidine to inhibit DNMT and reduce DNA methylation can regulate the anti-proliferative property of MUPCDH in HRCE and WT9-7 PKD cell lines, making it a potential therapeutic target (36). Apart from DNMT, specific inhibitors targeting HMTs, such as Smyd2 and EZH2, with their inhibitors delayed cyst growth in Pkd1 mutant mouse kidneys (12, 48). Since epigenetic modifying enzymes function in a wide range of organs in the body, more specific epigenetic treatment aiming to reverse the alterations occurring in PKD should lead to the development of treatment to reduce unwanted side effects, considering the complexity of methylation abnormalities occurring in different regions and genes on the PKD epigenome.

## Author Contributions

The research idea and study design were devised by ZM and CM. LZ revised the manuscript. JX and CX were the primary authors of this manuscript. XW finished data collection and statistical analysis. All authors read and approved the final manuscript.

## Funding

This work was supported by the National Natural Science Foundation of China (82070705, 81770670, 81873595, and 32030025), the National Key Research and Development Program of China (2019YFA0802001), Shanghai Municipal Key Clinical Specialty (shslczdzk02503), Shanghai Science and Technology Talent Program (19YF1450300), and Research Projects of Shanghai Science and Technology Committee (17411972100).

## Conflict of Interest

The authors declare that the research was conducted in the absence of any commercial or financial relationships that could be construed as a potential conflict of interest.

## Publisher's Note

All claims expressed in this article are solely those of the authors and do not necessarily represent those of their affiliated organizations, or those of the publisher, the editors and the reviewers. Any product that may be evaluated in this article, or claim that may be made by its manufacturer, is not guaranteed or endorsed by the publisher.

## References

[1] Iglesias CG Torres VE Offord KP Holley KE Beard CM Kurland LT. Epidemiology of adult polycystic kidney disease, Olmsted County, Minnesota:1935-1980. Am J Kidney Dis. (1983) 2:630–9. 10.1016/S0272-6386(83)80044-4 6846334

[2] Collins AJ Foley RN Herzog C Chavers BM Gilbertson D Ishani A. Excerpts from the US renal data system 2009 annual data report. Am J Kidney Dis. (2010) 55(Suppl. 1): A426-7. 10.1053/j.ajkd.2009.10.009 20082919 PMC2829836

[3] Cornec-Le Gall E Audrezet MP Le Meur Y Chen JM Ferec C. Genetics and pathogenesis of autosomal dominant polycystic kidney disease: 20 years on. Hum Mutat. (2014) 35:1393–406. 10.1002/humu.22708 25263802

[4] Consortium TIPKD. Polycystic kidney disease:the complete structure of the PKD1 gene and its protein. Cell. (1995) 81:289–98. 10.1016/0092-8674(95)90339-9 7736581

[5] Mochizuki T Wu G Hayashi T Xenophontos SL Veldhuisen B Saris JJ. PKD2, a Gene for Polycystic Kidney Disease That Encodes an Integral Membrane Protein. Science. (1996) 272:1339-42. 10.1126/science.272.5266.1339 8650545

[6] Leonidas Tsiokas TA Chenwen ZHU Gerdwalz Vikas P. Sukhatme Specific association of the gene product of PKD2 with the TRPC1 channel. Proc Natl Acad Sci USA. (1999) 96:3934–9. 10.1073/pnas.96.7.3934 10097141 PMC22398

[7] Harris PC Rossetti S. Molecular diagnostics for autosomal dominant polycystic kidney disease. Nat Rev Nephrol. (2010) 6:197–206. 10.1038/nrneph.2010.18 20177400 PMC4050432

[8] Y P. Nature and nurture on phenotypic variability of autosomal dominant polycystic kidney disease. Kid Int. (2005) 67:1630–31. 10.1111/j.1523-1755.2005.00252.x 15780121

[9] Milutinovic J Rust PF Fialkow PJ Agodoa LY Phillips LA Rudd TG. Intrafamilial phenotypic expression of autosomal dominant polycystic kidney disease. Am J Kidney Dis. (1992) 19:465–72. 10.1016/S0272-6386(12)80956-5 1585936

[10] Li X. Epigenetics and autosomal dominant polycystic kidney disease. Biochim Biophys Acta. (2011) 1812:1213–8. 10.1016/j.bbadis.2010.10.008 20970496 PMC3413450

[11] Li X. Epigenetics in ADPKD: understanding mechanisms and discovering treatment. In: Li X, editor. Polycystic Kidney Disease. Brisbane, QLD: Codon Publications (2015). 27512785

[12] Li X. Epigenetics and cell cycle regulation in cystogenesis. Cell Signal. (2020) 68:109509. 10.1016/j.cellsig.2019.109509 31874209 PMC8154103

[13] Egger G Liang G Aparicio A Jones PA. Epigenetics in human disease and prospects for epigenetic therapy. Nature. (2004) 429:457–63. 10.1038/nature02625 15164071

[14] Kenter AT Rentmeester E van Riet J Boers R Boers J Ghazvini M. Cystic renal-epithelial derived induced pluripotent stem cells from polycystic kidney disease patients. Stem Cells Transl Med. (2020) 9:478–90. 10.1002/sctm.18-0283 32163234 PMC7103626

[15] Ramalingam H Kashyap S Cobo-Stark P Flaten A Chang CM Hajarnis S. A methionine-Mettl3-N(6)-methyladenosine axis promotes polycystic kidney disease. Cell Metab. (2021) 33:1234–1247. 10.1016/j.cmet.2021.03.024 33852874 PMC8172529

[16] McCabe MT Davis JN Day ML. Regulation of DNA methyltransferase 1 by the pRb/E2F1 pathway. Cancer Res. (2005) 65:3624–32. 10.1158/0008-5472.CAN-04-2158 15867357

[17] Deaton AM Bird A. CpG islands and the regulation of transcription. Genes Dev. (2011) 25:1010–22. 10.1101/gad.2037511 21576262 PMC3093116

[18] Bowden SA Rodger EJ Chatterjee A Eccles MR Stayner C. Recent discoveries in epigenetic modifications of polycystic kidney disease. Int J Mol Sci. (2021) 22:13327. 10.3390/ijms222413327 34948126 PMC8708269

[19] Marc Jung WX. Swati Kadam,Tibor A. MIRA-seq for DNA methylation analysis of CpG islands. Epigenomics. (2015) 7:695–706. 10.2217/epi.15.33 25881900 PMC4607651

[20] Jiang CL Jin SG Pfeifer GP. MBD3L1 is a transcriptional repressor that interacts with methyl-CpG-binding protein 2 (MBD2) and components of the NuRD complex. J Biol Chem. (2004) 279:52456–64. 10.1074/jbc.M409149200 15456747

[21] Rauch T Li H Wu X Pfeifer GP. MIRA-assisted microarray analysis, a new technology for the determination of DNA methylation patterns, identifies frequent methylation of homeodomain-containing genes in lung cancer cells. Cancer Res. (2006) 66:7939–47. 10.1158/0008-5472.CAN-06-1888 16912168

[22] Rauch TA Pfeifer GP. The MIRA method for DNA methylation analysis. Methods Mol Biol. (2009) 507:65–75. 10.1007/978-1-59745-522-0_6 18987807 PMC2824570

[23] Bowden SA Rodger EJ Bates M Chatterjee A Eccles MR Stayner C. Genome-Scale single nucleotide resolution analysis of DNA methylation in human autosomal dominant polycystic kidney disease. Am J Nephrol. (2018) 48:415–24. 10.1159/000494739 30463078

[24] Chatterjee A RE Morison IM Eccles MR Stockwell PA. Tools and strategies for analysis of genome-wide and gene-specific DNA methylation patterns. Methods Mol Biol. (2017) 1537:249–77. 10.1007/978-1-4939-6685-1_15 27924599

[25] M G-G M F. CpG islands in vertebrate genomes. J Mol Biol. (1987) 196:261–82. 10.1016/0022-2836(87)90689-9 3656447

[26] Adusumalli S Mohd Omar MF Soong R Benoukraf T. Methodological aspects of whole-genome bisulfite sequencing analysis. Brief Bioinform. (2015) 16:369–79. 10.1093/bib/bbu016 24867940

[27] Nicoglou A Merlin F. Epigenetics: A way to bridge the gap between biological fields. Stud Hist Philos Biol Biomed Sci. (2017) 66:73–82. 10.1016/j.shpsc.2017.10.002 29033228

[28] Jin Z Liu Y DNA. methylation in human diseases. Genes Dis. (2018) 5:1–8. 10.1016/j.gendis.2018.01.002 30258928 PMC6147084

[29] Reddington JP Sproul D Meehan RR. DNA methylation reprogramming in cancer: does it act by re-configuring the binding landscape of Polycomb repressive complexes? BioEssays. (2014) 36:134–40. 10.1002/bies.201300130 24277643 PMC4225474

[30] Ke Sun DX. Changlin Mei. The association between autosomal dominant polycystic kidney disease and cancer. Int Urol Nephrol. (2019) 51:93–100. 10.1007/s11255-018-1951-5 30109558

[31] Woo YM Bae J-B Oh Y-H Lee Y-G Lee MJ Park EY. Genome-wide methylation profiling of ADPKD identified epigenetically regulated genes associated with renal cyst development. Hum Genet. (2013) 133:281–97. 10.1007/s00439-013-1378-0 24129831

[32] Hajirezaei F Ghaderian SMH Hasanzad M Nafar M Ghadiani MH Biglari S. Methylation of the PKD1 promoter inversely correlates with its expression in autosomal dominant polycystic kidney disease. Rep Biochem Mol Biol. (2020) 9:193–8. 10.29252/rbmb.9.2.193 33178869 PMC7603252

[33] Bowden SA Stockwell PA Rodger EJ Parry MF Eccles MR Stayner C. Extensive inter-cyst DNA methylation variation in autosomal dominant polycystic kidney disease revealed by genome scale sequencing. Front Genet. (2020) 11:348. 10.3389/fgene.2020.00348 32351541 PMC7174623

[34] Bu L. The role of abnormal DNA methylation inautosomal dominant polycystic kidney disease and its mechanism [D] Wangfang Database. (2019).

[35] Hansen KD Timp W Bravo HC Sabunciyan S Langmead B McDonald OG. Increased methylation variation in epigenetic domains across cancer types. Nat Genet. (2011) 43:768–75. 10.1038/ng.865 21706001 PMC3145050

[36] Woo YM Shin Y Hwang JA Hwang YH Lee S Park EY. Epigenetic silencing of the MUPCDH gene as a possible prognostic biomarker for cyst growth in ADPKD. Sci Rep. (2015) 5:15238. 10.1038/srep15238 26463459 PMC4604459

[37] Bronkhorst AJ Ungerer V Holdenrieder S. The emerging role of cell-free DNA as a molecular marker for cancer management. Biomol Detect Quantif. (2019) 17:100087. 10.1016/j.bdq.2019.100087 30923679 PMC6425120

[38] Kim DY Woo YM Lee S Oh S Shin Y Shin JO. Impact of miR-192 and miR-194 on cyst enlargement through EMT in autosomal dominant polycystic kidney disease. FASEB J. (2019) 33:2870–84. 10.1096/fj.201800563RR 30332302

[39] Bartel DP. MicroRNAs: genomics, biogenesis, mechanism, and function. Cell. (2004) 116:281–97. 10.1016/S0092-8674(04)00045-5 14744438

[40] Levenson VV DNA. methylation as a universal biomarker. Expert Rev Mol Diagn. (2010) 10:481–8. 10.1586/erm.10.17 20465502 PMC2933138

[41] Swarup V Rajeswari MR. Circulating (cell-free) nucleic acids–a promising, non-invasive tool for early detection of several human diseases. FEBS Lett. (2007) 581:795–9. 10.1016/j.febslet.2007.01.051 17289032

[42] Sawan C Herceg Z. Histone modifications and cancer. Adv Genet. (2010) 70:57–85. 10.1016/B978-0-12-380866-0.60003-4 20920745

[43] Zhang Y Reinberg D. Transcription regulation by histone methylation: interplay between different covalent modifications of the core histone tails. Genes Dev. (2001) 15:2343–60. 10.1101/gad.927301 11562345

[44] KDIGO Clinical Practice Guidelines for Glomerulonephritis. Chapter 3: Steroid-sensitive nephrotic syndrome in children. Kidney Int Suppl. (2012) 2:163-171. 10.1038/kisup.2012.16 25028636 PMC4089737

[45] Huang J Perez-Burgos L Placek BJ Sengupta R Richter M Dorsey JA. Repression of p53 activity by Smyd2-mediated methylation. Nature. (2006) 444:629–32. 10.1038/nature05287 17108971

[46] Cho HS Hayami S Toyokawa G Maejima K Yamane Y Suzuki T. RB1 methylation by SMYD2 enhances cell cycle progression through an increase of RB1 phosphorylation. Neoplasia. (2012) 14:476–86. 10.1593/neo.12656 22787429 PMC3394190

[47] Li LX Zhou JX Wang X Zhang H Harris PC Calvet JP et al. Cross-talk between CDK4/6 and SMYD2 regulates gene transcription, tubulin methylation, and ciliogenesis. Sci Adv. (2020) 6:eabb3154. 10.1126/sciadv.abb3154 33127671 PMC7608814

[48] Li LX Fan LX Zhou JX Grantham JJ Calvet JP Sage J. Lysine methyltransferase SMYD2 promotes cyst growth in autosomal dominant polycystic kidney disease. J Clin Invest. (2017) 127:2751–64. 10.1172/JCI90921 28604386 PMC5490754

[49] Plotnikova OV Pugacheva EN Golemis EA. Primary cilia and the cell cycle. Methods Cell Biol. (2009) 94:137–60. 10.1016/S0091-679X(08)94007-3 20362089 PMC2852269

[50] Sherr CJ Beach D Shapiro GI. Targeting CDK4 and CDK6: from discovery to therapy. Cancer Discov. (2016) 6:353–67. 10.1158/2159-8290.CD-15-0894 26658964 PMC4821753

[51] Vandel L Nicolas E Vaute O Ferreira R Ait-Si-Ali S Trouche D. Transcriptional repression by the retinoblastoma protein through the recruitment of a histone methyltransferase. Mol Cell Biol. (2001) 21:6484–94. 10.1128/MCB.21.19.6484-6494.2001 11533237 PMC99795

[52] Fan LX Li X Magenheimer B Calvet JP Li X. Inhibition of histone deacetylases targets the transcription regulator Id2 to attenuate cystic epithelial cell proliferation. Kidney Int. (2012) 81:76–85. 10.1038/ki.2011.296 21900881 PMC3409467

[53] Zhou X Fan LX Sweeney WE. Jr. Denu JM Avner ED Li X. Sirtuin 1 inhibition delays cyst formation in autosomal-dominant polycystic kidney disease J Clin Invest. (2013) 123:3084–98. 10.1172/JCI64401 23778143 PMC4101988

[54] Kuzmichev A Nishioka K Erdjument-Bromage H Tempst P Reinberg D. Histone methyltransferase activity associated with a human multiprotein complex containing the Enhancer of Zeste protein. Genes Dev. (2002) 16:2893–905. 10.1101/gad.1035902 12435631 PMC187479

[55] Cao R Wang L Wang H Xia L Erdjument-Bromage H Tempst P. Role of histone H3 lysine 27 methylation in Polycomb-group silencing. Science. (2002) 298:1039–43. 10.1126/science.1076997 12351676

[56] Bracken AP Pasini D Capra M Prosperini E Colli E Helin K. EZH2 is downstream of the pRB-E2F pathway, essential for proliferation and amplified in cancer. EMBO J. (2003) 22:5323–35. 10.1093/emboj/cdg542 14532106 PMC213796

[57] Nachtergaele S He C. Chemical Modifications in the Life of an mRNA Transcript. Annu Rev Genet. (2018) 52:349–72. 10.1146/annurev-genet-120417-031522 30230927 PMC6436393

[58] Bokar JA Shambaugh ME Polayes D Matera AG Rottman FM. Purification and cDNA cloning of the AdoMet-binding subunit of the human mRNA (N6-adenosine)-methyltransferase. RNA. (1997) 3:1233–47. 9409616 PMC1369564

[59] Jia G Fu Y He C. Reversible RNA adenosine methylation in biological regulation. Trends Genet. (2013) 29:108–15. 10.1016/j.tig.2012.11.003 23218460 PMC3558665

[60] Padovano V Podrini C Boletta A Caplan MJ. Metabolism and mitochondria in polycystic kidney disease research and therapy. Nat Rev Nephrol. (2018) 14:678–87. 10.1038/s41581-018-0051-1 30120380

[61] Jones PA Baylin SB. The epigenomics of cancer. Cell. (2007) 128:683–92. 10.1016/j.cell.2007.01.029 17320506 PMC3894624

